# Origin, genomic diversity and evolution of African swine fever virus in East Asia

**DOI:** 10.1093/ve/vead060

**Published:** 2023-10-07

**Authors:** Genyang Xin, Qiyuan Kuang, Shijia Le, Weichen Wu, Qi Gao, Han Gao, Zhiying Xu, Zezhong Zheng, Gang Lu, Lang Gong, Heng Wang, Guihong Zhang, Mang Shi, Yankuo Sun

**Affiliations:** State Key Laboratory for Biocontrol, School of Medicine, Shenzhen campus of Sun Yat-sen University, Shenzhen 518107, China; Guangdong Provincial Key Laboratory of Zoonosis Prevention and Control, College of Veterinary Medicine, South China Agricultural University, Guangzhou 510642, PR China; African Swine Fever Regional Laboratory of China (Guangzhou), South China Agricultural University, Guangzhou 510642, PR China; Research Center for African Swine Fever Prevention and Control, South China Agricultural University, Guangzhou 510642, PR China; Maoming Branch, Guangdong Laboratory for Lingnan Modern Agriculture, Guangdong 510642, PR China; State Key Laboratory for Biocontrol, School of Medicine, Shenzhen campus of Sun Yat-sen University, Shenzhen 518107, China; State Key Laboratory for Biocontrol, School of Medicine, Shenzhen campus of Sun Yat-sen University, Shenzhen 518107, China; Guangdong Provincial Key Laboratory of Zoonosis Prevention and Control, College of Veterinary Medicine, South China Agricultural University, Guangzhou 510642, PR China; African Swine Fever Regional Laboratory of China (Guangzhou), South China Agricultural University, Guangzhou 510642, PR China; Research Center for African Swine Fever Prevention and Control, South China Agricultural University, Guangzhou 510642, PR China; Maoming Branch, Guangdong Laboratory for Lingnan Modern Agriculture, Guangdong 510642, PR China; Guangdong Provincial Key Laboratory of Zoonosis Prevention and Control, College of Veterinary Medicine, South China Agricultural University, Guangzhou 510642, PR China; African Swine Fever Regional Laboratory of China (Guangzhou), South China Agricultural University, Guangzhou 510642, PR China; Research Center for African Swine Fever Prevention and Control, South China Agricultural University, Guangzhou 510642, PR China; Maoming Branch, Guangdong Laboratory for Lingnan Modern Agriculture, Guangdong 510642, PR China; Guangdong Provincial Key Laboratory of Zoonosis Prevention and Control, College of Veterinary Medicine, South China Agricultural University, Guangzhou 510642, PR China; African Swine Fever Regional Laboratory of China (Guangzhou), South China Agricultural University, Guangzhou 510642, PR China; Research Center for African Swine Fever Prevention and Control, South China Agricultural University, Guangzhou 510642, PR China; Maoming Branch, Guangdong Laboratory for Lingnan Modern Agriculture, Guangdong 510642, PR China; Guangdong Provincial Key Laboratory of Zoonosis Prevention and Control, College of Veterinary Medicine, South China Agricultural University, Guangzhou 510642, PR China; African Swine Fever Regional Laboratory of China (Guangzhou), South China Agricultural University, Guangzhou 510642, PR China; Research Center for African Swine Fever Prevention and Control, South China Agricultural University, Guangzhou 510642, PR China; Maoming Branch, Guangdong Laboratory for Lingnan Modern Agriculture, Guangdong 510642, PR China; Guangdong Provincial Key Laboratory of Zoonosis Prevention and Control, College of Veterinary Medicine, South China Agricultural University, Guangzhou 510642, PR China; African Swine Fever Regional Laboratory of China (Guangzhou), South China Agricultural University, Guangzhou 510642, PR China; Research Center for African Swine Fever Prevention and Control, South China Agricultural University, Guangzhou 510642, PR China; Maoming Branch, Guangdong Laboratory for Lingnan Modern Agriculture, Guangdong 510642, PR China; Guangdong Provincial Key Laboratory of Zoonosis Prevention and Control, College of Veterinary Medicine, South China Agricultural University, Guangzhou 510642, PR China; African Swine Fever Regional Laboratory of China (Guangzhou), South China Agricultural University, Guangzhou 510642, PR China; Research Center for African Swine Fever Prevention and Control, South China Agricultural University, Guangzhou 510642, PR China; Maoming Branch, Guangdong Laboratory for Lingnan Modern Agriculture, Guangdong 510642, PR China; Guangdong Provincial Key Laboratory of Zoonosis Prevention and Control, College of Veterinary Medicine, South China Agricultural University, Guangzhou 510642, PR China; African Swine Fever Regional Laboratory of China (Guangzhou), South China Agricultural University, Guangzhou 510642, PR China; Research Center for African Swine Fever Prevention and Control, South China Agricultural University, Guangzhou 510642, PR China; Maoming Branch, Guangdong Laboratory for Lingnan Modern Agriculture, Guangdong 510642, PR China; Guangdong Provincial Key Laboratory of Zoonosis Prevention and Control, College of Veterinary Medicine, South China Agricultural University, Guangzhou 510642, PR China; African Swine Fever Regional Laboratory of China (Guangzhou), South China Agricultural University, Guangzhou 510642, PR China; Research Center for African Swine Fever Prevention and Control, South China Agricultural University, Guangzhou 510642, PR China; Maoming Branch, Guangdong Laboratory for Lingnan Modern Agriculture, Guangdong 510642, PR China; State Key Laboratory for Biocontrol, School of Medicine, Shenzhen campus of Sun Yat-sen University, Shenzhen 518107, China; State Key Laboratory for Biocontrol, School of Medicine, Shenzhen campus of Sun Yat-sen University, Shenzhen 518107, China; Guangdong Provincial Key Laboratory of Zoonosis Prevention and Control, College of Veterinary Medicine, South China Agricultural University, Guangzhou 510642, PR China; African Swine Fever Regional Laboratory of China (Guangzhou), South China Agricultural University, Guangzhou 510642, PR China; Research Center for African Swine Fever Prevention and Control, South China Agricultural University, Guangzhou 510642, PR China; Maoming Branch, Guangdong Laboratory for Lingnan Modern Agriculture, Guangdong 510642, PR China

**Keywords:** ASFV, African swine fever virus, metagenome, evolution, genetic diversity

## Abstract

Since 2018, the outbreaks of genotype II African swine fever virus (ASFV) in China and several eastern Asian countries have caused a huge impact on the local swine industry, resulting in huge economic losses. However, little is known about the origin, genomic diversity, evolutionary features, and epidemiological history of the genotype II ASFV. Here, 14 high-quality complete genomes of ASFVs were generated via sequencing of samples collected from China over the course of 3 years, followed by phylogenetic and phylodynamic analyses. The strains identified were relatively homogeneous, with a total of 52 SNPs and 11 indels compared with the prototype strain HLJ/2018, among which there were four exceptionally large deletions (620–18,023 nt). Evolutionary analyses revealed that ASFV strains distributed in eastern Asia formed a monophyly and a ‘star-like’ structure centered around the prototype strain, suggesting a single origin. Additionally, phylogenetic network analysis and ancestral reconstruction of geographic state indicated that genotype II ASFV strains in eastern Asia likely originated from Western Europe. Overall, these results contribute to the understanding of the history and current status of genotype II ASFV strains in eastern Asian, which could be of considerable importance in disease control and prevention.

## Introduction

African swine fever (ASF) caused by African swine fever virus (ASFV) is a highly contagious hemorrhagic disease that affects domestic and wild pigs of all ages, with a mortality rate that can be as high as 100 per cent (Zhao et al. 2019). ASF has caused tremendous losses to the global pig industry, with no effective vaccine against the virus. Since pork meat account for >35 per cent of the global meat intake, the virus poses a serious problem to food security worldwide (WOAH 2023).

ASFV is the sole member of the family *Asfarviridae* with a double-stranded DNA genome of ∼170–193 kb in length and is comprised of 151–186 open reading frames (ORFs) (Bao et al. 2019). Based on sequence conservation, the genome can be roughly divided into a left variable region (LVR) of 1–16 kb, a conserved central region (CCR) of ∼125 kb, and a right variable region (RVR) of 38–47 kb in length (Blasco, et al. 1989; Sumption, et al. 1990; Irusta, et al. 1996; Bao, et al. 2019). Based on the partial nucleotide Sequence of the C-terminal end of the gene B646L, which encodes the major capsid protein (MCP) P72, ASFV strains are classified into 24 different genotypes (Bastos et al. 2003; Njau et al. 2021). All of the field strains from outside Africa belong to genotypes I and II (Sauter-Louis, et al. 2021; Penrith, et al. 2022).

The first case of ASF was reported in Kenya in the 1920s, and since then it has been reported in 32 African countries and 74 countries across the rest of the world (Montgomery 1921; Penrith, et al. 2022; WOAH 2023). In China, the first case of ASF was reported in a suburb of Shenyang, Liaoning province in 2018, with acute clinical and pathological symptoms, such as high fever, dullness, and skin redness, and all pigs died within 1 month (Zhou, et al. 2018; Liu, et al. 2020). Subsequently, ASF outbreaks was reported in both domestic pigs and wild pigs in 31 provinces (Jia, et al. 2020; Liu, et al. 2020), and ASFV was detected in neighboring countries, including South Korea, India, Cambodia, Mongolia, Vietnam, and Laos. Analysis of P72 gene of genomes isolated from infected pigs revealed that the ASFV detected during that outbreak was genotype II (Ge, et al. 2018; Zhou, et al. 2018; Bao, et al. 2019; Zhao, et al. 2019). The first report of genotype II ASFV was confirmed based on the analysis of the strain China/SY/2018 (ASFV/SY18) identified from the earliest reported case during the outbreak. Additionally, the first ASFV isolation was carried out based on a sample obtained in Heilongjiang province of China (China/HLJ/2018), which was used to characterize the virulence, replication features, and complete genomic sequence of the virus (Ge, et al. 2018; Wen, et al. 2019; Zhao, et al. 2019). Further genetic analysis revealed that the China/HLJ/2018 strain was similar to the Georgia/2007 and PoL/2017 (Pol17_04461_C210) strains; therefore, it was speculated to be of eastern European or Russia origin (Bao et al. 2019; Wen et al. 2019).

Similar to other dsDNA viruses, ASFV genome is highly conserved, and nucleotide substitution the main driving force of genomic evolution occurs at extremely slow rate (Shen et al. 2022). Additionally, other driving forces that shape the diversity of ASFV are deletions and insertions, which tend to appear at the two ends of the genomes LVR and RVR. Approximately 70 per cent of indels (insertions–deletions) were not longer than 10 bp, with only ∼9 per cent barely more than 50 bp (Blasco et al. 1989; Zhu et al. 2019). Particularly, indels were more frequent in the 5ʹ-end of the genome. In the genotype II AFSV, several genomic variations are used to distinguish strains from different geographic locations. For example, the 10 bp tandem repeat in IGR I73R/I329L is often used to identify eastern Europe and Asia strains (named IGR II genotype), tandem repeat in MGF 505–9 R/10 R is used to identify Poland strains, and variations in B602 L and O174L genes are used to identify Estonia and Poland strains (Mazur-Panasiuk and Woźniakowski 2019; Mason-D’Croz et al. 2020; Mazur-Panasiuk et al. 2020; Zhenzhong et al. 2022). Moreover, large deletions were detected in LVR (Sun et al. 2022).

Although genotype II ASFV outbreak has been devastating in China and neighboring countries in Asia, there is a lack of information of its origin, genomic diversity, evolutionary features, and epidemiological history (Ge, et al. 2018; Zhou, et al. 2018). Here, we filled in the knowledge gap here via sequencing of samples collected from 14 swine production site in China and performed in-depth phylodynamic analyses. It is anticipated that the findings of this study would contribute to the understanding of the emergence, spread, and evolution of the genotype II ASFV in eastern Asia.

## Materials and methods

### Sample collection and processing

Samples were collected from 14 swine production sites at Guangdong, Guangxi, Jiangxi, and Hunan provinces of China. Pigs in these sites experienced typical symptoms of ASF, including fever, anorexia, dyspnea, emesis, constipation, and dermorrhagia, followed by ∼90–100 per cent death with 6–15 days (Zhao et al. 2019). Tissues, including blood, were obtained from diseased pigs and preserved in ice packs until they were sent to laboratory at Guangzhou, China. Total nucleic acid was extracted from the blood samples using the AxyPrep^TM^ Body Fluid Viral DNA/RNA Miniprep Kit (Axygen, the USA). One microliter of nucleic acid was then subject to real-time PCR using the AceQ universal U1 probe master mix V2 (Vazyme, China) with primers and probe targeting the B646L gene of ASFV (F:ATAGAGATACAGCTCTTCCAG, R: GTATGTAAGAGCTGCAGAAC, Prob: FAM-TATCGATAAGATTGAT-MGB). The qPCR assays were performed on the QuantStudioTM 5 (Thermo, the USA) platform, and the ASFV-positive samples with qPCR C*t* value < 26 were subject to DNA library construction and sequencing.

### Next-generation sequencing library construction and sequencing

DNA libraries were constructed using Nextera XT DNA Library Prep Kit (Illumina, the USA). Library quality and concentration were determined using Qseq1 (Bioptic Inc., China) and Qubit (Life technologies, the USA), respectively. Samples that failed the library quality control step were prepared using VAHTS Universal Plus DNA Library Prep Kit for Illumina (Vazyme, China), which target low-concentration and highly degraded samples. A 150-bp pair-end sequencing was performed on the Novaseq 6000 (Illumina, the USA) platform.

### Genome assembly and characterization

Quality control of raw reads, including the discard of reads with average quality below 10, adaptor sequences, and reads shorter than 90 nt, was performed using bbduk program in BBMap package (https://sourceforge.net/projects/bbmap/). Subsequently, genome assembly was performed using both *de novo* assembly and genomic mapping approaches. *De novo* genome assembly were performed using SPAdes program version 3.15.2 (Prjibelski et al. 2020) under—meta and -k 21,33,55,77 parameter settings, which conduct metagenomic data in a series of k-mer. Genomic mapping was performed using bowtie2 v2.3.5.1 (Langmead and Salzberg 2012) under the ‘end-to-end’ mode (namely,—phred33—end-to-end—sensitive—no-unal), with the genome sequence of ASFV strain China/HLJ/2018 (Accession number: NC_044959.2) as the template. The final genome sequence of each ASFV strain was derived based on the results of both *de novo* and mapping assembly approaches. Single-nucleotide polymorphisms (SNPs) and indels were determined by comparing against the China/HLJ/2018 strain. A 5-fold coverage threshold was used for SNP calling. And an indel is considered as false positive if it appears within a repeating unit with > 5 nt. All variations identified were subsequently confirmed by PCR/Nested-PCR and Sanger sequencing. The PCR/Nested-PCR were performed using Taq PCR StarMix (GenStar, China) based on paired primers (Supplementary Table S1) designed using Oligo7 and Primer-BLAST (https://www.ncbi.nlm.nih.gov/tools/primer-blast/index.cgi?LINK_LOC=BlastHome), followed by Sanger sequencing.

### Genome alignment

A total of 50 high-quality reference complete or near complete genomes of genotype II ASFV were downloaded from the National Center for Biotechnology Information Nucleotide Database under the taxonomy ‘taxid10497’ (Supplementary Table S2), and compared against the sequences obtained in this study. Sequence alignments were performed using the fast Fourier transform (FFT) based progressive method FFT-NS-2 algorithm implemented in a FFT based multiple sequence alignment program MAFFT version 7.475 (Rozewicki et al. 2019), followed by manual alignment curation to correct ambiguously aligned regions and repeat regions. Columns with less than 70 per cent sequence information were removed from the genotype II ASFV multiple sequence alignment using Trimal (Capella-Gutiérrez, Silla-Martínez, and Gabaldón 2009).

### Phylogenetic analyses

Phylogenetic trees were reconstructed using both maximum likelihood and Bayesian approaches. Phylogenetic tree based on maximum likelihood was constructed on PhyML version 20120412 software (Guindon et al. 2010) using General Time Reversible (GTR) nucleotide substitution model and Subtree Pruning and Regrafting (SPR) branch-swapping algorithms Phylogenetic tree based on Bayesian approach was constructed on MrBayes version 3.2.7 (Ronquist et al. 2012), using GTR model with gamma-distributed rate variation across sites and a proportion of invariable sites. The analysis was run for 1,000,000 generations and sampled every 1000 generations, with the initial 25 per cent discarded as burn-in.

### Inference of geographical expansion history of ASFVs in China

All sampling locations associated with ASFV sequences used here were grouped into six regions, Africa, Central Europe, Western Europe, Eastern Europe, Western Asia, and Eastern Asia, according to their geographic locations. Two approaches were used to determine the geographic expansion history. First, an ancestral state reconstruction approach was implemented using Mesquite program version 3.70 (Maddison 2008). In this approach, we estimated the most likely geographic location at each well-supported ancestral node in the phylogeny, based on the MCMC tree generated using the MrBayes software (see Phylogenetic analyses) and a Markov *k*-state 1 parameter (Mk1, a maximum-likelihood reconstruction method) model. Additionally, a phylogenetic network approach was used to infer the lineage expansion history of ASFV using DnaSP program version 6.12.03 (Rozas, et al. 2017), and the haplotype network was shown using TCS network implemented in PopART (Leigh and Bryant 2015). Genomes with large deletions were not included in the analyses to avoid potential loss of phylogenetic informative sties.

## Results

### Characterization and comparisons of ASFV genomes identified in China

**Figure 1.**
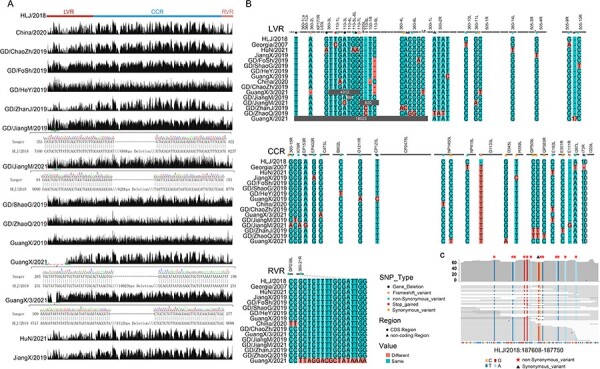
Characterization and comparisons of genotype II ASFV genomes identified in this study. (A) mapping of sequencing reads against prototype strain China/HLJ/2018. The confirmation results of major gaps using PCR and Sanger sequencing are show under the genomes GD/JiangM/2019, GD/JiangM/2021, GuangX/2021, and GuangX/3/2021. (B) Characterization of SNPs and indels for genomes identified in this study in comparisons with China/HLJ/2018. Variations that occurred in coding regions are represented with solid circles, whereas those in non-coding regions are represented with sold triangles. The color of circles and triangles represent type of SNPs. The 10-bp insertion shown in the figure represents ‘GTTAAGTCAATAGTTTA’ insertion in comparisons to China/HLJ/2018 strain and 17bp insertion preresent ‘TATATAGGAA’ in comparisons to China/HLJ/2018 strain. (C) Mapping reads from sample GuangX/2021 against China/HLJ/2018 confirms the hypovriable region between site 615 and 700 of 360-21R gene. Variations from the reference sequence are shown in different colors, synonymous changes are represented with black triangles, and non-synonymous changes are represented with red solid stars.

A total of 14 genome sequences of genotype II ASFV were generated from clinically ill domestic pig in China (Fig. 1A). Although 20 samples were sequenced, only 14 yielded > 10-fold average sequence depth (Table 1), which was considered as adequate coverage for subsequent genomic confirmation and analyses. The total number of reads ranged from 4.2 to 10 Gbps per sample, which resulted in 14–369 folds sequencing depth (median 103) and > 99 per cent coverage of the prototype type II ASFV genome identified in China (China/HLJ/2018). Following PCR confirmation, the final genome length ranged from 172,771 to 190,791 bp, which was similar to that of the China/HLJ/2018 strain, with the exception of GuangX/2021 (172,771 bp) and GuangX/3/2021 (185,531 bp) strains, which had relatively shorter genome due to large proportions of deletions near the 5ʹ-end (Supplementary Appendix, Fig. S1; Fig. 1B, Table 1). Additionally, there were variations in 4–28 nt among viruses identified in the present study, and 2–18 nt differences compared with the prototype Chinese strain China/HLJ/2018 (Supplementary Appendix, Fig. S1).

**Table 1.** Information on 14 Samples.

Sample name	Collect City	Collect time	Coverage rate (%)	Mean Coverage	Process
GD/JiangM/2021	JiangMen/GuangDong	2021	99.7	27	Vazyme
HuN/2021	HuNan	2021	99.97	369	Nextera XT
JiangX/2019	JiangXi	2019	99.996	170	Nextera XT
GD/FoSh/2019	FoShan/GuangDong	2019	99.98	352	Nextera XT
GD/ShaoG/2019	ShaoGuang/GuangDong	2019	99.9	30	Nextera XT
GD/HeY/2019	HeYuang/GuangDong	2019	99.99	23	Vazyme
GuangX/2019	GuangXi	2019	99.999	22	Nextera XT
China/2020	China	2020	99.9	150	Nextera XT
GD/ChaoZh/2019	ChaoZhou/GuangDong	2019	99.99	229	Nextera XT
GuangX/3/2021	GuangXi	2021	97.8	211	Vazyme
GD/JiangM/2019	JiangMen/GuangDong	2019	99.9	241	Vazyme
GD/ZhanJ/2019	ZhanJiang/GuangDong	2019	99.4	57	Nextera XT
GD/ZhaoQ/2019	ZhaoQing/GuangDong	2019	99.95	14	Vazyme
GuangX/2021	GuangXi	2021	90.9	203	Nextera XT

Reference Genome: MK333180.1 ASFV China/HLJ/2018.

Vazyme: VAHTS Universal Plus DNA Library Prep Kit for Illumina.

Nextera XT: Nextera XT DNA Library Prep Kit.

Moreover, substitutions were identified in the newly identified ASFV genomes compared with the genome of the China/HLJ/2018 strain. A total of 52 SNPs were identified, including 43 located in 26 protein-coding genes and nine in non-coding regions. Among the SNPs located within the genes, 29 were non-synonymous changes. Interestingly, although most of the genes contained a maximum of two substitutions, 360–21 R gene in the RVR contained a total of 16 substitutions, including 14 non-synonymous ones, all of which occurred in the GuangX/2021 strain. Indeed, each substitution was supported by >60-folds coverage, suggesting that it was not likely due to sequencing error. Additionally, a single non-synonymous change (strain JiangX/2019) was identified in the EP402R gene-encoding transmembrane protein CD2v, which induced the replacement of Glu with Lys at protein position 247. Specifically, this protein is responsible for the hemadsorption of red blood cells around infected cells (Goatley and Dixon 2011). Overall, all Chinese variants contained the signature substitution A/G at position 986 in 360–10 L gene, a major difference between the Chinese strain China/HLJ/2018 and its closest relatives from Europe.

Furthermore, four insertions and seven deletions were identified in the present study (Fig. 1A). Specifically, PCR confirmed four major deletions in LVR, including the 18023, 4312, 688, and 620 nt deletions in GuangX/2021, GuangX/3/2021, GD/JiangM/2019, and GD/JiangM/2021, respectively, which resulted in loss of 1–32 genes in MGFs or ACDs (Fig. 1A and B). Two single-nucleotide deletions were identified in the MGF 110–7 L gene (position 37 and 47) and one single-nucleotide deletion in the MGF 360–2L gene (position 242), which resulted in frameshift in affected genes. Moreover, four insertions were found in LVR and CCR, with one located in non-coding regions. Specifically, a 17-bp tandem repeat insertion occurred between MGF 360–9 R and MGF 360–10 R genes in LVR. The remaining insertions were single-nucleotide insertions in the coding regions of the genes 110–7 L (position 292), D1133L (position 3125), and E111R (position 257), causing frameshift in the three genes.

### Diversity and evolutionary history of genotype II ASFV in China

**Figure 2.**
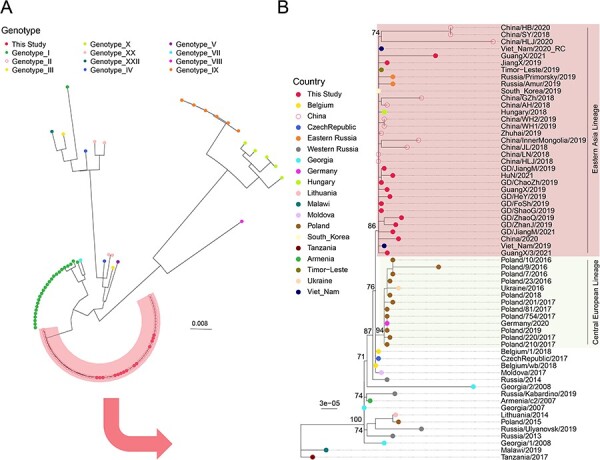
Phylogenetic analyses of ASFV genomes identified in this study. (A) A phylogeny involves all representative ASFV strains. Different genotypes are marked with different color. Viruses identified in this study are represented by solid red circles. (B) A phylogeny involves only genotype II ASFV genomes. Support of the phylogenetic tree topology is only shown on internal nodes. Viruses from different geographic regions are marked with different color. Two well-supported lineages, namely, Eastern Asia Linage and Central European Lineage, were highlighted with red and yellow background colors.

Phylogenetic analysis was performed to determine the diversity of the ASFVs identified in the present. All 14 strains of viruses identified belonged to genotype II ASFV (Fig. 2A), and were distinctly clustered within a well-supported monophyletic lineage tentatively named ‘Eastern Asia Lineage’ comprising of strains identified in China (*N* = 26) and neighboring regions, including Vietnam (*N* = 2), eastern Russia (*N* = 2), South Korea (*N* = 1), and Timor-Leste (*N* = 1). A single Hungarian virus strain was also found in this lineage.

**Figure 3.**
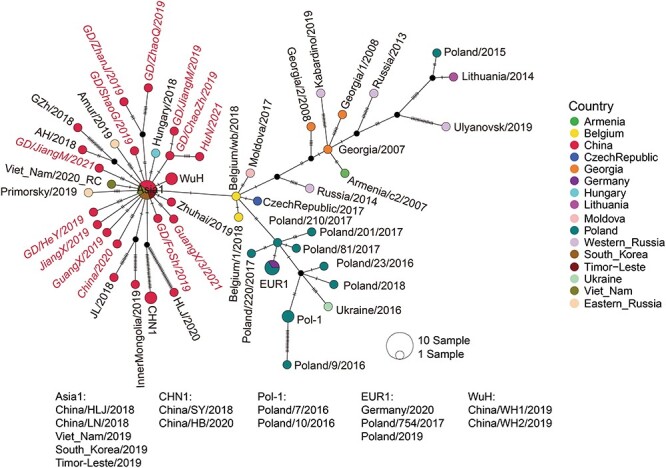
Phylogenetic network analyses reveal the geographic origin of genotype II ASFV circulating in Eastern Asia. Echo circle represent a strain and the black circle represents a hypothetical strain. Size of circles represents the numbers of gnomes included, the color in the circle represents different country or regions, and the number of striping on the line between two circles represents the number of SNPs. The sequences reported in this study are annotated in red.

Additionally, the entire ‘Eastern Asia Lineage’ shared close relationship with viruses from several eastern and western Europe countries, and the closest strain was the Belgian strain Belgium/wb/2018 with only one nucleotide difference compared with the China/HLJ/2018 strain (Fig. 3; Supplementary Appendix, Fig. S1), followed by Belgium/1/2018 and CzechRepublic/2017, which contained two-nucleotide differences compared with the China/HLJ/2018 strain. Moreover, a group of viruses from mainly eastern European countries (Poland and Ukraine) were related to the Eastern Asia Lineage but shared more distant relationship with the Chinese strain China/HLJ/2018, with 3–21 nt differences.

Furthermore, majority of the viruses within the Eastern Asia Lineage shared close relationship with each other and with the China/HLJ/2018 strain (<5 nt differences), and there was no obvious geographic clustering of strains at both regional (within China) or country level (Fig. 2B). In addition to those closely related to the prototype strain China/HLJ/2018, the Eastern Asia Lineage also contain several more divergent strains, such as GuangX/2021 (identified and confirmed in the present study), China/HB/2020, China/SY/2018, and China/HLJ/2020, sharing 0–25 nt variations compared with the China/HLJ/2018 strain. Overall, these viruses contained unique SNPs or large deletions that were distinct from one another, suggesting that they were more locally defined.

### Inference of the origin and spread of the Eastern Asia Lineage

Phylogenetic network analyses were performed to reveal the dynamics of genotype II ASFV (Fig. 3, Supplementary Table S2). The network showed that the Eastern Asia Lineage expanded owing to a single outbreak, with several ancestral viral genomes occupying the central nodes (namely ‘Asia1’ node, which contained China/HLJ/2018, China/LN/2018, amongst others) along with various types of mutated descendant genomes (Fig. 3). Importantly, the outbreak was linked to Belgium/wb/2018, which shared only a single-nucleotide variation with the ancestral ‘Asia1’ node (A/G in 360–10 L gene). Interestingly, Belgium/wb/2018 and related strains were also the ‘intermediary’ genetic variant between the Eastern Asia Lineage and central (mainly Poland strains) and eastern (mainly Russian and Georgia strains) European lineages (Fig. 3). Within the Eastern Asia Lineage, the ancestral ‘Asia1’ node was shared by strains sampled from China, Vietnam, South Korea, and Timor-Leste. Since the earliest isolate was identified in China, the strains from east and southeast Asian countries were most likely to have originated from China. Similarly, later sampling of mutated variants of this lineage in Russia and Hungary were also traced back to the 2018 AFSV outbreak in China (Fig. 3).

**Figure 4.**
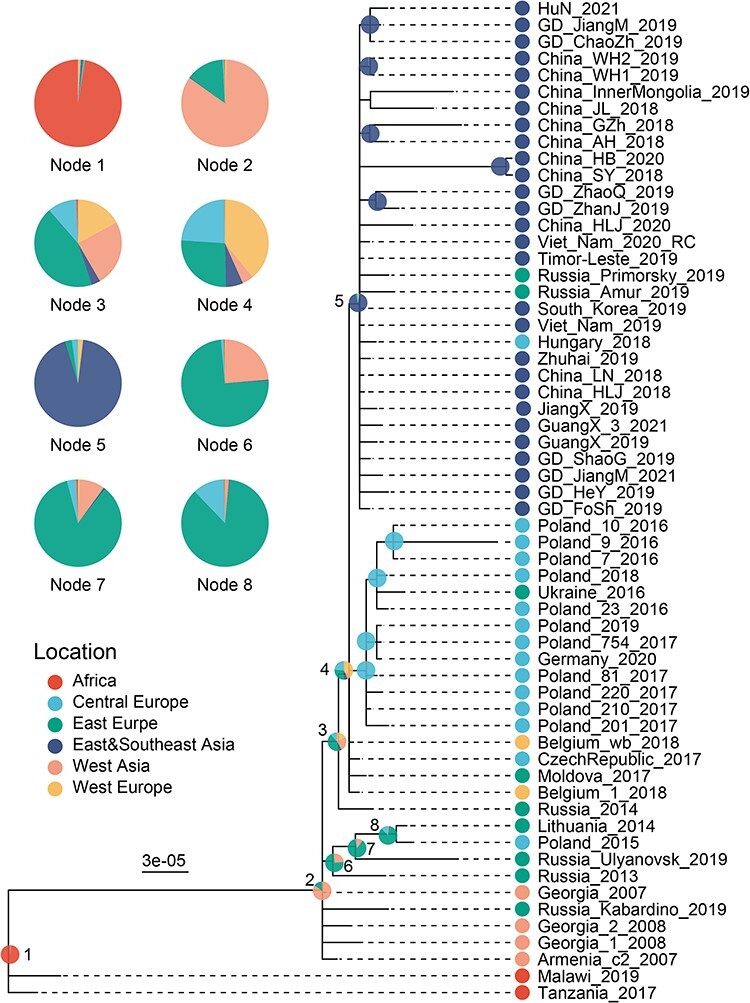
Reconstruction of ancestral geographic regions for genotype II ASFV. Phylogenetic tree was reconstructed using MrBays and ancestral reconstruction of geographic regions was carried out in Mesquite. A consensus tree is shown here with result from ancestral geographic reconstructions (presented in colored pie chart) marked on each of the ancestral nodes. The proportion of area in pie chart represent the probability support for each geographic region in that node.

In addition to phylogenetic network analyses, ancestral reconstruction was performed to reveal the most likely geographic region, including Central Europe, Eastern Europe, Eastern Asia, Western Asia, or Western Europe, for each of the internal node in the phylogenetic tree to enable the construction of the viral dispersal pathway (Fig. 4). The ancestral location for the entire Eastern Asia Lineage (Node 5) was most likely in Eastern Asia (*P* = 0.93), and therefore the Russian and Hungarian strains that branched from Node 5 most likely originated from Eastern Asia. Additionally, the ancestral region for Node 4 was estimated to be more likely in Western Europe (*P* = 0.39) than in Eastern (*P* = 0.31) and Central Europe (*P* = 0.22) based on current sampling, suggesting a potential Western European origin for AFSV outbreak in East Asia; however, further studies are necessary to confirm this speculation.

### Genomic evolution of genotype II ASFV

**Figure 5.**
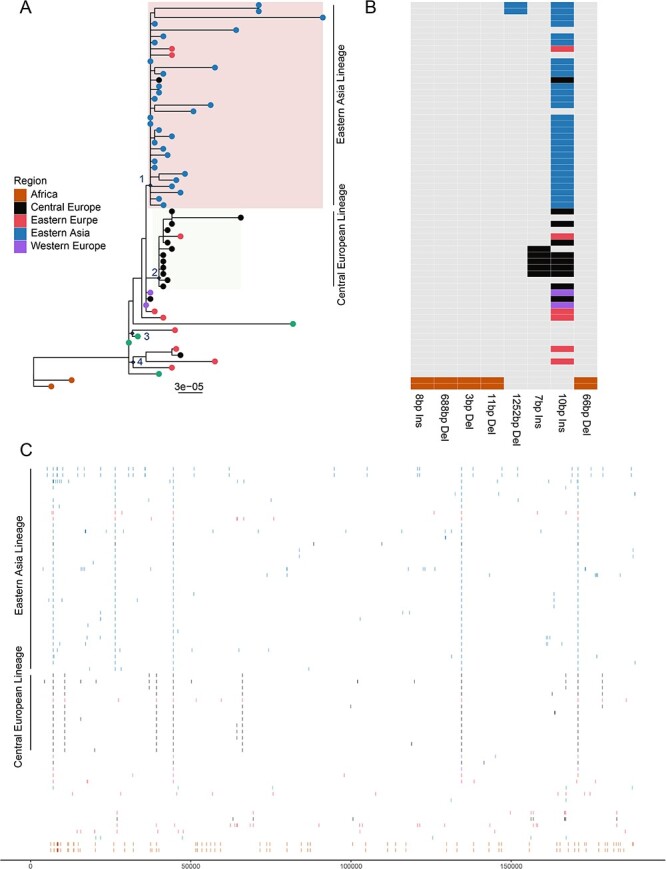
Identification of SNPs and Indel markers in genotype II ASFV genomes, all sequences are on the same order. (A) A phylogeny involves only genotype II ASFV genomes, the numbers on the tree represent import nodes. (B) Indels longer than 3 nt and substitution represented by >2 genomes are showed. (C) SNPs relative to strain Georgia/2007. The colors in the tree, indel plot, and SNP plot represent different geographic regions.

Furthermore, we traced the evolutionary history of the entire genotype II ASFV and identified potential markers for the Eastern Asia Lineage and other important lineages. Indels appeared frequently in ASFV genomes, but they were highly sporadic and only few of them were consistent with the phylogenetic structure defined based on nucleotide substitutions (Fig. 5). For example, a few indels can be used to distinguish African and non-African genotype II ASFV, including 10 and 8 bp insertions, and 3, 11, and 688 bp deletions. Additionally, a group of Poland strains within Central Europe Lineage had a unique set of 7 bp insertion in O174R gene. Moreover, a 10-nt tandem repeat insertion between I73R and I329L genes were widely but not exclusively observed in the Eastern Asia Lineage (31/33) and Central Europe Lineage (9/15). The 10-nt tandem repeat insertion was also observed in several earlier divergent Russian, Armenian, and Georgian strains, but with lower frequencies (2/8).

Regarding nucleotide substitutions, the single signature substitution that defined the Eastern Asia Lineage was A/G at position 986 in 360–10 L gene (Node 1, Fig. 5). Additionally, the Central Europe Lineage was defined by three substitutions, including two G/A (site 10,668 and 39,306 on Georgia/2007) and C/A (site 66,152 on Georgia/2007) (Node 2, Fig. 5). Moreover, the Eastern Asian Lineage, Central Europe Lineage, and a few western European strains were distinguished from earlier eastern European and western Asian strains by substitutions, including C/T (site 7059 on Georgia/2007), A/G (site 44,576 on Georgia/2007), T/C (site 134,514 on Georgia/2007), and T/A (site 170,862 on Georgia/2007), which defined nodes 3 and 4. Furthermore, the African and non-African type II ASFVs were distinguished by 72 substitutions distributed along ASFV genome (Fig. 5).

## Discussion

In the present study, 14 complete genomes of ASFVs were generated via sequencing of samples collect in China from 2019 to 2021. Phylodynamic analysis indicated that the recent circulation of ASFVs in China and neighboring countries (the Eastern Asia Lineage) resulted from a single outbreak, followed by rapid dispersal across China, sporadic transmissions to other countries, and localized spread and endemic status within China. This conclusion was based on the finding that the current diversity was ‘star-like’, with all strains centered around the ancestral type or prototype strain (i.e. China/HLJ/2018); moreover, no further large-scale transmission chains have been established from any of the new mutant variants. Overall, these observations indicate that the ASFVs have been ‘stabilized’ or localized four years after the initial outbreak in 2018 (Zhou, et al. 2018; Bao, et al. 2019; Shen, et al. 2022).

Phylogenetic network analysis to determine the origin of the Eastern Asia Lineage showed that the Belgian strain Belgium/wb/2018 was the closest variant, with only a single-nucleotide variation compared with the prototype strain. Specifically, the virus was detected in a dying wild boar located in *Bois de Buzenol* which in the south of Belgium close to the borders to France and Luxembourg (Gilliaux et al. 2019). Although the closest relative was identified from Belgium (Western Europe), geographic reconstructions of ancestral nodes revealed that the origin of the Eastern Asia Lineage might also be Central and Eastern Europe, but with lower probability. This is because other (more distant) close relatives were mainly from central and eastern European countries, making these regions potential origin as well. Therefore, two likely scenarios were proposed for the origin of the Eastern Asia Lineage based on the evidence provided in the present study. The most likely scenario is that the lineage originated from central or eastern Europe and transmitted to China via a western European country. Alternatively, the lineage might have been directly transmitted from central or eastern Europe to China; however, further evidence that these regions harbored viruses identical to either China/HLJ/2018 or Belgium/wb/2018 prior to the 2018 outbreak is necessary to support this assumption.

Furthermore, a total of 52 SNPs, seven deletions, and four insertions were identified in the strains sequenced in the present study compared with the prototype strain China/HLJ/2018 (Wen et al. 2019). Although majority of the viruses shared the same genomic features and high genetic identity (<5 nt differences), there were several exceptions. For instance, four viruses had large deletions (620–18,023 nt) in the LVR, which resulted in loss of several functional genes mainly located in the multigene family 100, 110, and 360. However, the exact function of these genes remains unclear, although deletion of the entire multigene family in ASFV is linked to reduced virulence in cell cultures (Yanez et al. 1995; Wang et al. 2021). Moreover, large deletions in the LVR have been reported in the previously identified strain ASFV_YNFN202103 (Sun et al. 2022), with 2,144 nt and 7,772 nt deletions, suggesting that deletions at LVR were normal and not rare. In addition to deletion, 16 substitutions were detected within a range of 86 nt located in the 360–21 R gene in the hyper-variant strain GuangX/2021, although the underlying genetic mechanism remained unclear.

In addition to genotype II ASFV, there were also reports of genotype I and genotype I and II recombinant strains circulating in China in 2021 and 2022, with increasing prevalence rates (Sun et al. 2021; Zhao et al. 2023). Evolutionary analysis of China’s genotype I ASFV was showed to share high genetic similarity with strains NH/P68 and OURT 88/3 (Sun et al. 2021). However, these strains are not detected in this study, most likely due to the timing and geographic regions of our sampling. Nevertheless, given the endemic status and homogeneous population of genotype II ASFV, it is highly likely that the new variants, namely, the genotype I and the recombinant strains, will become more prevalent or even cause new waves of outbreaks in China and Eastern Asia.

Our study identified a hypervariable region, which is characterized by 16 substitutions detected within a range of 86 nt located in the 360–21 R gene. Similar hypervariable region was also identified from ASFV strains China/HLJ/2020, where a total of 25 SNPs were detected in gene MGF 110–1 L, although these cannot be independently verified. One potential mechanism, as proposed for monkeypox virus, was APOBEC3-induced mutation. In this mechanism, APOBEC3 enzyme can be upregulated during viral infection and can inhibit virus replication by introducing mutations through deaminase and deaminase-independent mechanisms (Sadeghpour et al. 2021; Isidro et al. 2022; Pecori et al. 2022). These mutations are often directional and in rare cases viruses might survive such mutagenesis and become highly divergent from parental strains. For example, the human APOBEC3 caused G > A and C > T mutations in HPV genome (Vartanian et al. 2008; Isidro et al. 2022). However, the ASFV hypervariable region in GuangX/2021 and China/HLJ/2020 had no such mutation preference (A:3, C:5, G:6, T:5 in GuangX/2021 and A:7, C:6, G:9, T:5 in China/HLJ/2020), which indicates that the APOBEC3 may not account for the occurrence of these mutations. Alternatively, it might be due to recombination between divergent strains of ASFV, although the genomic origin of that minor recombined region remains unclear.

## Supplementary Material

vead060_Supp

## Data Availability

The ASFV genomic sequences generated in this study have been deposited in the CNCB GenBase database under the accession C_AA040127.1 ∼ C_AA040140.1. The raw sequencing reads have been deposited in the SRA database under the project accession PRJNA1007294.
