# Supplementary material for: Origin, genomic diversity and evolution of African swine fever virus in East Asia
Source: Virus Evol. 2023 Oct 7;9(2):vead060. doi: 10.1093/ve/vead060 (PMC10590196; doi:10.1093/ve/vead060)
Supplement: vead060_Supp [file vead060_supp.zip › vead060_Supp/ST1.docx]

**Supplementary Table 1 PCR Primers used in this study**

| Forward PCR primer | Reverse PCR primer | Type | Description |
| --- | --- | --- | --- |
| CATATACGGTGTTGCGGGCT | GGTTGCGTTGCATTTGCGAT | Paired | Refgenome 14100 to 14300 for Ploy-C Region |
| AGTTCTCTCATAGGAGGCGT | ATTGTTTTGCCTTAGGCGCAG | Paired | Refgenome 15500 to 16500 for Ploy-C Region |
| ACCTATGGGTCCGTTTTCCC | TACGGCCTTAGAGACGTTGA | Paired | Refgenome 17600 to 18000 for Gaps/lower Coverage region of China/2020 |
| TTCGTCCTTTCCGTCTTGGC | GTCAGCAAACCATAACAATGCTAA | Paired | Refgenome 19800 to 2000 for Gaps/lower Coverage region of China/2020, GD/FoSh/2020 and GD/JiangM/2021 |
| GTTACGGCAAAAGAGCCGTG | AATTTCACGGCTATCGGGGT | Paired | Refgenome 21400 to 21900 for Gaps/lower Coverage region of HuN/2021 |
| AACACTGGCTGGCTATCGTC | GGCTTAACGCCTGTATCCGA | Paired | Refgenome 32700 to 32900 for Gaps/lower Coverage region of GD/JiangM/2021 |
| CGACTCATGAGAGTGGACCT | GTCGCCCTCTAAAGCTCCTA | Paired | Primers for 17 bps Insert of GuangX/2021 |
| ACATAGCTACTTCCCTAAGCAGT | TGGAAGTGGTGTCATCATGC | Paired | Refgenome 73900 to 74800 for Gaps/lower Coverage region of GuangX/2021 |
| CTGCTTTTCCACATGTAGCT |  | Single | Medium Sequencing Primer for PCR product of  Refgenome 73900 to 74800 |
| TTTTAGGCGTGTCAGCCTGT | CCTAAAGCGTCTTCCGCAGT | Paired | Primers for 10 bps insertion between I73R and I329L |
| CATAACTGGTGTTACGCCGC | TGGACACCAGTGAACGTGT | Paired | Primers for 18023 bps Deletion of GuangX/2021 |
| CGAGGAAGTGTCTGTTGGAGTC | GCTAGTCGTTGCTAGCCGT | Paired | Primers for 4312 bps Deletion of GuangX/3/2021 |
| ATCGTGCATTCTGCCTGGAA | TGCCAAGACGGCACTTGTAT | Paired | Outer primers for 620 bps Deletion of GD/JiangM/2021 |
| CTCTTCCAGGGGATTGTCGG | GGGTGAGTCACTTGGTTTGC | Paired | Inner primers for 620 bps Deletion of GD/JiangM/2021 |
| ATACAAGTGCCGTCTTGGCAG | ACTACCAACCCGTTATCCTCCT | Paired | Primers for 688 bps Deletion of GD/JiangM/2020 |
| Reference Genome: NC_044959.2 ASFV Georgia 2007/1  All Productions were Sequencing using PCR Primers. | | | |
