# Supplementary material for: Origin, genomic diversity and evolution of African swine fever virus in East Asia
Source: Virus Evol. 2023 Oct 7;9(2):vead060. doi: 10.1093/ve/vead060 (PMC10590196; doi:10.1093/ve/vead060)
Supplement: vead060_Supp [file vead060_supp.zip › vead060_Supp/Supplementary Table 2 Genotype II ASFV genomes used in this study.docx]

**Supplementary Table 2 Genotype II ASFV genomes used in this study**

| Accession | Strain name | Name in this study | Reference |
| --- | --- | --- | --- |
| LR812933.1 | Arm/07/CBM/c2 | Armenia/c2/2007 | Pérez-Núñez D, et al. 2020 |
| LR536725.1 | Belgium_2018/1 | Belgium/1/2018 | Pikalo, et al. 2020 |
| MK543947.1 | Belgium/Etalle/wb/2018 | Belgium/wb/2018 | Gilliaux, et al. 2019 |
| MK645909.1 | ASFV/wbBS01 | China/JL/2018 | Unpublished |
| MT496893.1 | GZ201801 | China/GZh/2018 | Unpublished |
| MK333181.1 | DB/LN/2018 | China/LN/2018 | Wen, et al. 2019 |
| MK333180.1 | Pig/HLJ/2018 | China/HLJ/2018 | Wen, et al. 2019 |
| MK940252.1 | CN/2019/InnerMongolia/AES01 | China/InnerMongolia/2019 | Unpublished |
| MW361944.1 | China/GD/2019 | China/GD/2019 | Wang, et al. 2022 |
| MN393476.1 | Wuhan_2019/1 | China/WH1/2019 | Xiong, et al. 2021 |
| MN393477.1 | Wuhan_2019/2 | China/WH2/2019 | Xiong, et al. 2021 |
| MW521382.1 | HuB20 | China/HB/2020 | Unpublished |
| MW656282.1 | Pig/Heilongjiang/HRB1/2020 | China/HLJ/2020 | Sun, et al. 2021 |
| MK128995.1 | China/2018/AnhuiXCGQ | China/AH/2018 | Bao, et al. 2019 |
| MH766894.2 | ASFV/SY18 | China/SY/2018 | Unpublished |
| LR722600.1 | CzechRepublic_2017/1 | CzechRepublic/2017 | Forth, et al. 2020 |
| LS478113.1 | Estonia_2014 | Estonia/2014 | Vilem, et al 2020 |
| NC_044959.2 | Georgia_2007/1 | Georgia/2007 | Chapman, et al. 2011 |
| MH910496.1 | Georgia_2008/2 | Georgia/2/2008 | Farlow, et al. 2018 |
| MH910495.1 | Georgia_2008/1 | Georgia/1/2008 | Farlow, et al. 2018 |
| LR899193.1 | Germany_2020/1 | Germany/2020 | Sauter‐Louis, et al. 2021 |
| MN715134.1 | HU_2018 | Hungary/2018 | Olasz, et al. 2019 |
| MK628478.1 | ASFV/LT14/1490 | Lithuania/2014 | Gallardo, et al. 2017 |
| MW856068.1 | MAL/19/Karonga | Malawi/2019 | Hakizimana, et al. 2020 |
| LR722599.1 | Moldova_2017/1 | Moldova/2017 | Forth, et al. 2019 |
| MH681419.1 | ASFV/POL/2015/Podlaskie | Poland/2015 | Olesen, et al. 2018 |
| MG939586.1 | Pol16_29413_o23 | Poland/23/2016 | Mazur-Panasiuk, et al. 2019 |
| MT847622.1 | Pol17_31177_O81 | Poland/81/2017 | Mazur-Panasiuk, et al. 2020 |
| MT847620.1 | Pol17_55892_C754 | Poland/754/2017 | Mazur-Panasiuk, et al. 2020 |
| MG939589.1 | Pol17_05838_C220 | Poland/220/2017 | Mazur-Panasiuk, et al. 2019 |
| MG939588.1 | Pol17_04461_C210 | Poland/210/2017 | Mazur-Panasiuk, et al. 2019 |
| MG939587.1 | Pol17_03029_C201 | Poland/201/2017 | Mazur-Panasiuk, et al. 2019 |
| MG939585.1 | Pol16_20540_o10 | Poland/10/2016 | Mazur-Panasiuk, et al. 2019 |
| MG939584.1 | Pol16_20538_o9 | Poland/9/2016 | Mazur-Panasiuk, et al. 2019 |
| MG939583.1 | Pol16_20186_o7 | Poland/7/2016 | Mazur-Panasiuk, et al. 2019 |
| MT847621.1 | Pol18_28298_O111 | Poland/2018 | Mazur-Panasiuk, et al. 2020 |
| MT847623.2 | Pol19_53050_C1959/19 | Poland/2019 | Mazur-Panasiuk, et al. 2020 |
| KJ747406.1 | Kashino 04/13 | Russia/2013 | Varentsova, et al. 2015 |
| KP843857.1 | Odintsovo 02/14 | Russia/2014 | Varentsova, et al. 2015 |
| MT459800.1 | ASFV/Kabardino/Balkaria | Russia/Kabardino/2019 | Mazloum, et al. 2021 |
| MW306192.1 | ASFV/Ulyanovsk_19/WB/5699 | Russia/Ulyanovsk/2019 | Mazloum, et al. 2021 |
| MW306191.1 | ASFV/Primorsky_19/WB/6723 | Russia/Primorsky/2019 | Mazloum, et al. 2021 |
| MW306190.1 | ASFV/Amur_19/WB/6905 | Russia/Amur/2019 | Mazloum, et al. 2021 |
| LR813622.1 | Tanzania/Rukwa/2017/1 | Tanzania/2017 | Njau, et al. 2021 |
| MW396979.1 | Timor-Leste-2019/1 | Timor-Leste/2019 | Mileto, et al. 2021 |
| MN194591.1 | Kyiv/2016/131 | Ukraine/2016 | Kovalenko, et al. 2019 |
| MT166692.1 | Hanoi_2019 | Viet_Nam/2019 | Tran, et al. 2021 |
| MW465755.1 | 05L1/HaNam/VN/2020 | Viet_Nam/2020 | Truong, et al. 2021 |
| MN172368.1 | China/CAS19-01/2019 | Zhuhai/2019 | Jia, et al. 2020 |
| MT748042.1 | Korea/pig/PaJu1/2019 | South_Korea/2019 | Kim, et al. 2020 |

**References**

Pérez-Núñez D, Castillo-Rosa E, Vigara-Astillero G, et al. Identification and isolation of two different subpopulations within african swine fever virus Arm/07 stock. Vaccines. 2020;8(4):625.

Pikalo J, Schoder ME, Sehl J, et al. The African swine fever virus isolate Belgium 2018/1 shows high virulence in European wild boar. Transboundary and Emerging Diseases. 2020;67(4):1654-1659.

Wang X, Wang X, Zhang X, et al. Genetic characterization and variation of African swine fever virus China/GD/2019 strain in domestic pigs. Pathogens. 2022;11(1):97.

Xiong D, Zhang X, Xiong J, et al. Rapid genome-wide sequence typing of African swine fever virus based on alleles. Virus Research. 2021;297:198357.

Sun E, Zhang Z, Wang Z, et al. Emergence and prevalence of naturally occurring lower virulent African swine fever viruses in domestic pigs in China in 2020. Science China Life Sciences. 2021;64(5):752-765.

Forth JH, Forth LF, Václavek P, et al. Whole-genome sequence of an African swine fever virus isolate from the Czech Republic. Microbiology Resource Announcements. 2020;9(44):e00948-20.

Vilem A, Nurmoja I, Niine T, et al. Molecular characterization of African swine fever virus isolates in Estonia in 2014–2019. Pathogens. 2020;9(7):582.

Chapman DA, Darby AC, Da Silva M, et al. Genomic analysis of highly virulent Georgia 2007/1 isolate of African swine fever virus. Emerging infectious diseases. 2011;17(4):599.

Farlow J, Donduashvili M, Kokhreidze M, et al. Intra-epidemic genome variation in highly pathogenic African swine fever virus (ASFV) from the country of Georgia. Virology journal. 2018;15(1):1-8.

Sauter‐Louis C, Forth JH, Probst C, et al. Joining the club: First detection of African swine fever in wild boar in Germany. Transboundary and Emerging Diseases. 2021;68(4):1744-1752.

Olasz F, Mészáros I, Marton S, et al. A simple method for sample preparation to facilitate efficient whole-genome sequencing of African swine fever virus. Viruses. 2019;11(12):1129.

Gallardo C, Soler A, Nieto R, et al. Experimental infection of domestic pigs with African swine fever virus Lithuania 2014 genotype II field isolate. Transboundary and Emerging Diseases. 2017;64(1):300-304.

Hakizimana J, Kamwendo G, Chulu J, et al. Genetic profile of African swine fever virus responsible for the 2019 outbreak in northern Malawi. BMC veterinary research. 2020;16(1):1-10.

Forth JH, Forth LF, King J, et al. A deep-sequencing workflow for the fast and efficient generation of high-quality African swine fever virus whole-genome sequences. Viruses. 2019;11(9):846.

Olesen AS, Lohse L, Dalgaard MD, et al. Complete genome sequence of an African swine fever virus (ASFV POL/2015/Podlaskie) determined directly from pig erythrocyte-associated nucleic acid. Journal of virological methods. 2018;261:14-16.

Mazur-Panasiuk N, Woźniakowski G, Niemczuk KJSr. The first complete genomic sequences of African swine fever virus isolated in Poland. Scientific reports. 2019;9(1):4556.

Varentsova A, Yelsukova A, Zinyakov N, et al. Genomic aberrations in dna of African Swine Fever Virus circulating in the territory of the Russian Federation. Veterinary Science Today. 2015 (4):57-60.

Mazloum A, van Schalkwyk A, Shotin A, et al. Comparative analysis of full genome sequences of African swine fever virus isolates taken from wild boars in Russia in 2019. Pathogens. 2021;10(5):521.

Njau EP, Domelevo Entfellner J-B, Machuka EM, et al. The first genotype II African swine fever virus isolated in Africa provides insight into the current Eurasian pandemic. Scientific Reports. 2021;11(1):13081.

Mileto P, da Conceição F, Stevens V, et al. Complete Genome Sequence of African Swine Fever Virus Isolated from a Domestic Pig in Timor-Leste, 2019. Microbiology Resource Announcements. 2021;10(26):e00263-21.

Kovalenko G, Ducluzeau A-L, Ishchenko L, et al. Complete genome sequence of a virulent African swine fever virus from a domestic pig in Ukraine. Microbiology resource announcements. 2019;8(42):e00883-19.

Tran HTT, Truong AD, Dang AK, et al. Circulation of two different variants of intergenic region (IGR) located between the I73R and I329L genes of African swine fever virus strains in Vietnam. Transboundary and Emerging Diseases. 2021;68(5):2693-2695.

Truong QL, Nguyen TL, Nguyen TH, et al. Genome sequence of a virulent African swine fever virus isolated in 2020 from a domestic pig in Northern Vietnam. Microbiology Resource Announcements. 2021;10(19):e00193-21.

Jia L, Jiang M, Wu K, et al. Nanopore sequencing of African swine fever virus. Science China Life Sciences. 2020;63:160-164.

Kim HJ, Cho KH, Lee SK, et al. Outbreak of African swine fever in South Korea, 2019. Transboundary and Emerging Diseases. 2020;67(2):473-475.
