# Supplementary figures and images for: Origin, genomic diversity and evolution of African swine fever virus in East Asia
Source: Virus Evol. 2023 Oct 7;9(2):vead060. doi: 10.1093/ve/vead060 (PMC10590196; doi:10.1093/ve/vead060)

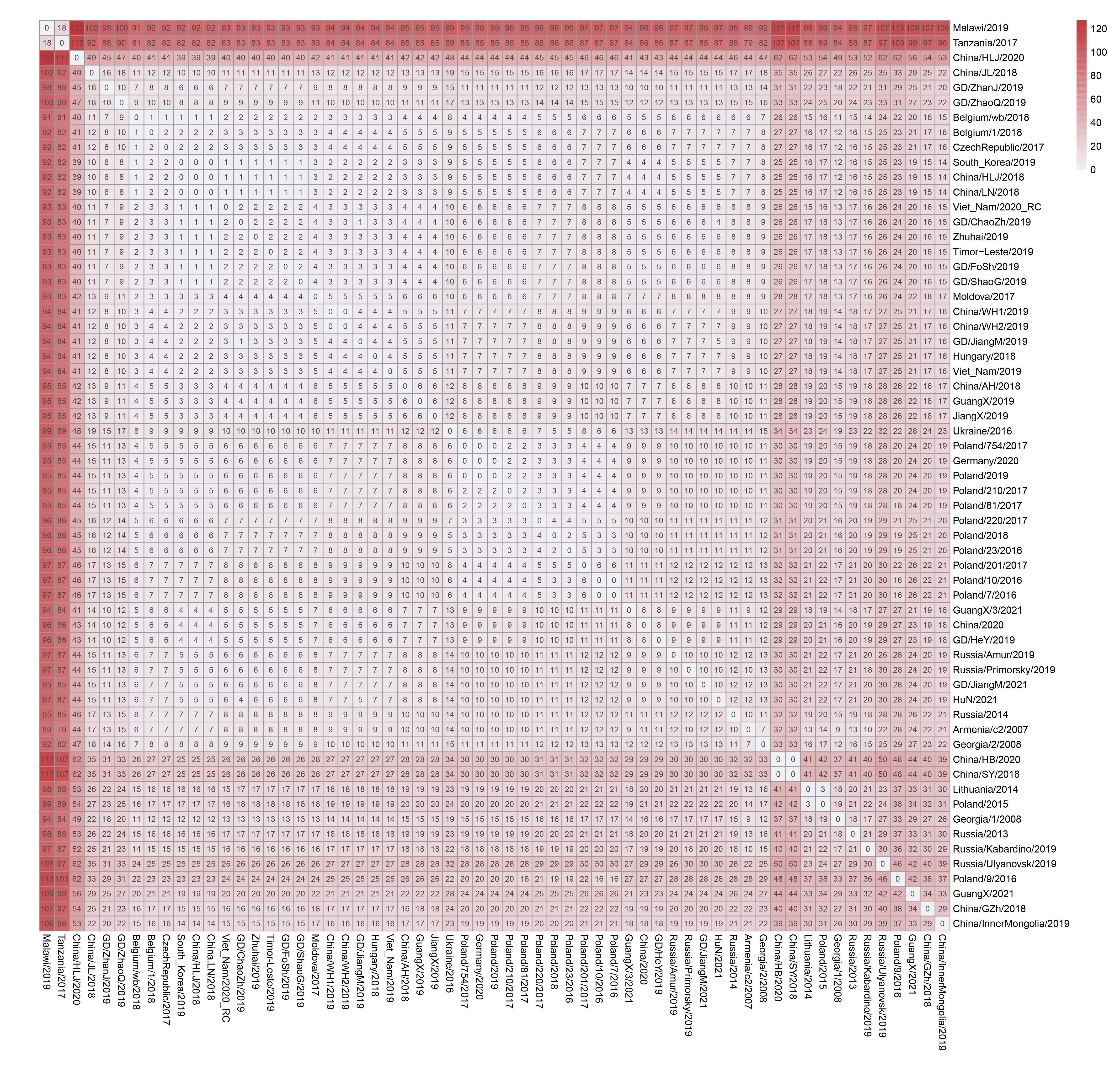

Supplement: vead060_Supp [file vead060_supp.zip › vead060_Supp/SFigure1.tif]
